# Development of a Web-Based Nomogram for Predicting Survival in Patients with Esthesioneuroblastoma Using SEER Database

**DOI:** 10.3390/cancers18193145

**Published:** 2026-09-29

**Authors:** Julianna L. Barbaro, Abhishek S. Bhutada, Srijan Adhikari, Kristine Ravina, Eric A. Marvin

**Affiliations:** 1 Virginia Tech Carilion School of Medicine, Virginia Tech, Roanoke, VA 24016, USA; asbhutada@carilionclinic.org (A.S.B.); sadhikari@carilionclinic.org (S.A.); kravina@carilionclinic.org (K.R.); eamarvin@carilionclinic.org (E.A.M.); 2 Translational Biology, Medicine, and Health Graduate Program, Fralin Biomedical Research Institute at Virginia Tech Carilion, Roanoke, VA 24016, USA; 3 Department of Neurosurgery, Carilion Clinic, Roanoke, VA 24014, USA

**Keywords:** esthesioneuroblastoma, Surveillance, Epidemiology, and End Result (SEER), overall survival, survival analysis, nomogram

## Abstract

**Background**: Esthesioneuroblastoma (ENB) is a rare neuroectodermal tumor arising from the olfactory epithelium. This study aimed to identify survival determinants and construct a prognostic web-based nomogram to provide individualized survival predictions for ENB patients. **Methods**: Patients diagnosed with ENB between 2000 and 2022 were identified using the Surveillance, Epidemiology, and End Results (SEER) database. Patient demographics, tumor characteristics, and treatment data were analyzed using Cox proportional hazards models and Kaplan–Meier survival curves. A web-based nomogram was constructed to predict 5- and 10-year survival probabilities individualized to a specific patient. **Results**: Nine hundred and forty-one ENB patients were analyzed. Overall survival (OS) rates at 1-, 2-, 5-, and 10-years were 89%, 85%, 75%, and 58%, respectively. Multivariable analysis revealed that age over 50 (*p* < 0.001) and tumor size over 6.0 cm (*p* < 0.01) were independent predictors of worse OS. Compared to patients treated with gross total resection (GTR) alone, worse OS was observed in those treated with chemotherapy (CT) only (*p* < 0.01), CT and radiation (RT, *p* < 0.01), GTR + CT (*p* < 0.05), RT only (*p* < 0.01), or no treatment (*p* < 0.001). No statistically significant differences in OS were found between GTR alone and GTR + RT, GTR + RT + CT, subtotal resection (STR), or STR with adjuvant therapies. The resulting ENB prognostic nomogram demonstrated acceptable predictive performance, with a C-index of 0.733 ± 0.041 and AUC of 0.749. **Conclusions**: Age and tumor size are critical survival determinants in ENB, and GTR continues to be the foundational treatment. The developed prognostic web-based nomogram successfully quantifies individualized survival probabilities, providing clinicians with a tailored tool to guide evidence-based counseling and management for ENB patients.

## Simple Summary

Esthesioneuroblastoma is a rare cancer that arises from the nerve cells in the nose. Considering this tumor is so uncommon, it can be challenging for doctors to know the optimal treatments for individual patients. In our study, we investigate medical information from 941 patients to understand how a person’s age, sex, race, tumor size and location, as well as treatments like surgery, chemotherapy and/or radiation affect their chances of survival. Using this data, we created an easy-to-use online tool called a nomogram. This clinical guide assists doctors and patients in predicting survival based on a person’s unique situation. Our findings will help the medical community by providing clearer guidance on how to predict patient outcomes. It further offers a helpful tool to improve personalized care and guide future research on this rare cancer.

## 1. Introduction

Esthesioneuroblastoma (ENB) is a sinonasal tumor also known as olfactory neuroblastoma that was first described in 1924 [1,2,3,4]. This tumor is of neuroectodermal origin, begins in basal progenitor cells of the olfactory epithelium near the cribriform plate, and accounts for 0.3% of upper aerodigestive tract cancers and 3–6% of all malignant nasal cavity tumors [2,3,4,5,6,7,8,9].

Symptoms are usually unilateral (as the tumor is unilateral) and are dependent on the location of the tumor within the nasal cavity. Common symptoms include: nasal obstruction, epistaxis with headaches, and hyposmia. Due to these symptoms mimicking infection, the diagnosis can be delayed 6–12 months [2,5,6,9,10]. Patients who present with sinonasal tumor symptoms are often referred to neurology/neurosurgery, ophthalmology and otolaryngology for a diagnostic work-up, as these patients could present with cranial nerve abnormalities (facial paresthesia, olfactory or ophthalmic deficits), middle ear effusion, proptosis or conjunctival injection [5].

Diagnosis is typically endoscopic with confirmatory histology/biopsy. Imaging (computerized tomography and magnetic resonance imaging) is also critical for initial evaluation, with lymph node fine needle aspiration used to assess for nodal spread [4,10]. Currently, clinicians characterize ENB by utilizing the Hyams histopathologic-based grading system which measures tumor cell aggressiveness, and the modified Kadish or Duguerov staging system which map the tumors’ physical extension into the skull or body. These grading and staging tools provide a tumor profile to guide treatment and predict survival in these patients [11,12,13]. ENB is particularly challenging to diagnose and distinguish from other sinonasal tumors due to (1) its low incidence (0.4 cases per million people) and few case reports (~1200), (2) its variable presentation, ranging from localized to metastatic at onset (17% of adults demonstrate lymph node metastasis at diagnosis) and (3) its lack of universal grading, staging and treatment [2,4,7]. A better understanding of prognostic factors that impact survival is warranted to help guide clinicians’ decision-making process for treatment.

Treatment of ENB can be done via surgery, radiation, and chemotherapy, alone or in combination. Given the location of the tumor near the orbit and anterior skull base, complete resection is not always possible. The most common treatment modality is surgery (anterior craniofacial resection) and post-operative radiation; the full benefit of radiation is not well defined. In unresectable or metastatic tumors, adjuvant chemotherapy is indicated, but some sources note that its benefit is unknown with others reserving it for palliative care options [2,3,5,8].

To our knowledge, this study represents the largest retrospective cohort to look at patients with ENB. Our objective is to utilize this large dataset to provide prognostic associations derived from a broader patient population. Here we propose an individualized prognostic model to estimate survival and guide clinical decision-making.

## 2. Materials and Methods

No Institutional Review Board approval was required because the data utilized in this dataset is publicly available. Our study complies with the National Cancer Institute’s (NCI) Surveillance, Epidemiology and End Result (SEER) database user agreement.

The SEER database, including 18 registries across the nation, was queried for patients diagnosed with ENB between 2000 and 2022. Patient demographics, tumor characteristics, and treatment data were analyzed using Cox proportional hazards (PH) models and Kaplan–Meier Curves (KMC). A web-based nomogram (https://abhibhutada.shinyapps.io/estapp/, accessed on 24 September 2026) was constructed to predict 5- and 10-year survival probabilities individualized to a specific patient.

### 2.1. Study Design and Cohort Selection

Data from 2000 to 2022 across 18 registries were gathered from the SEER database to retrospectively analyze patients diagnosed with ENB. The International Classification for Oncology, version 3 (ICD-O-3) histology code used was 9522/3 (Olfactory Neuroblastoma). The primary endpoint of this study was overall survival (OS) and is denoted by the time from diagnosis until death. To identify prognostic factors, univariate and multivariable Cox PH models were generated to analyze patient demographic, tumor, and treatment variables. The variables we chose to analyze were sex, age, race, year of diagnosis, tumor size, tumor location, and treatment summary. Any patients that did not have all the significant variables as determined by univariate analysis (sex, age, tumor location, tumor size, treatment) were removed from the multivariable analysis. Therefore, 483 patients were excluded due to missing tumor size. Furthermore, to avoid obscuring the understanding of our nomogram, we excluded 4 patients with tumors located outside the central nervous system (CNS) due to small sample size. However, 3 of these 4 patients were already excluded due to missing tumor size.

### 2.2. Statistical Analysis

Cox PH models were created with R (version 4.5.1, 2025) with “survival”, “survminer”, “ggplot2”, “readxl” and “rms” packages. All prognostic factors (sex, age, race, year of diagnosis, tumor location, tumor size, and treatment) had a univariate survival analysis conducted using Cox PH and are summarized as hazard ratios (HR), with 95% confidence intervals (CI).

Prognostic factors with statistically significant OS differences based on log-rank tests had KMCs generated. Statistically significant (*p* < 0.05) prognostic factors from our univariate analysis had individual KMCs generated to estimate survival. Furthermore, these significant variables were used to conduct a multivariable survival analysis. However, only patients with complete data (i.e., possessing all the significant variables) were included in the multivariable analysis.

Then, a web-based nomogram was built using the parametric survival modeling with Weibull accelerated failure time (AFT) to assess patient-specific ENB 5- and 10-year survival probability. Nomogram validity was assessed by calculating a concordance index and plotting a receiver operating characteristic (ROC) curve to obtain an area under the curve (AUC).

## 3. Results

### 3.1. Demographics

Prior to excluding patients with missing variables, 941 patients had ENB (Figure 1). There was a male (57%), white (65%) and ≥50 years old (61%) predominance. The mean age of diagnosis was 53.91 ± 16.82 YO. Based on the SEER-coded treatment categories, the tumor was most commonly located in the nasopharynx and nasal cavity (82%). Additionally, most patients received surgery (84%). Of the surgery patients, 31% received gross total resection (GTR) while 69% received subtotal resection (STR). Chemotherapy (CT) was given 70% of the time while radiotherapy (RT) was given 31% of the time. These percentages represented in Figure 1 demonstrate the number of patients receiving or not receiving a particular treatment, and do not investigate patients who received multi-modal treatment. Multimodal treatment is investigated in our univariate analysis.

### 3.2. Overall Survival (OS)

Our analysis of 941 patients demonstrated a median 13.6-year and mean 12.83-year OS with an 89.6% 1-year survival probability (Table 1).

### 3.3. Survival Analysis

Our patients had 1-, 2-, 3-, 5- and 10-year survival probabilities of 89.6%, 84.7%. 80.3%. 74.7% and 58.2%, respectively (Table 1). The univariate analysis included 941 patients investigating sex, age (0–50 years old and 50–100 years old), race (white, Hispanic, American Indian/Alaska Native [AI/AN], Asian or Pacific Islander [API], black), year of diagnosis (2000–2010 and 2010–2020), tumor location (nasopharynx and nasal cavity, CNS, and paranasal sinus), tumor size (<3, 3.0–6.0, >6.0 cm), and treatment summary (GTR, STR, CT and RT combinations) (Table 2).

OS was statistically significantly impacted by sex, age, tumor size, tumor location, and treatment. Male patients had a worse OS than female patients (hazard ratio [HR]: 1.36; 95% confidence interval [95% CI]: 1.10–1.68; *p* < 0.001). Patients who were ≥50 years old had a worse OS compared to those <50 years old (HR: 2.5; 95%CI: 1.98–3.16; *p* < 0.001). Patients with a tumor size 3–6 cm (HR: 1.65; 95%CI: 1.08–2.51; *p* < 0.05) and >6 cm (HR: 2.47; 95%CI: 1.51–4.03; *p* < 0.001) had a significantly worse OS than patients with a tumor size <3 cm. Patients with a paranasal sinus tumor (HR: 0.69; 95%CI: 0.52–0.93; *p* < 0.05) had a significantly greater OS compared to patients with a tumor in the nasopharynx and nasal cavity. Patients who received CT (HR: 10.7, 95%CI: 4.49–25.7; *p* < 0.001), CT + RT (HR: 4.33; 95%CI: 2.38–7.90; *p* < 0.001), RT (HR: 7.51; 95%CI: 3.52–16.0; *p* < 0.001), STR + CT (HR: 4.47; 95%CI: 1.87–10.7; *p* < 0.001) or no treatment (HR: 4.49; 95%CI: 2.43–8.28; *p* < 0.001) had significantly worse OS than patients who received GTR alone. Moreover, patients who received GTR + CT (HR: 6.76; 95%CI: 1.94–23.6; *p* < 0.01) and STR + CT + RT (HR: 2.05; 95%CI: 1.14–3.68; *p* < 0.05) had a significantly worse OS than patients who received GTR alone (Table 2).

Our multivariable analysis included sex, age, tumor size, tumor location and treatment. After excluding 483 patients for missing tumor size, and four patients with tumor location outside the CNS due to low sample size (three of the four patients were already excluded for missing tumor size), 457 patients were included in our multivariable analysis. To prevent duplication of variables, we excluded surgery, RT and CT from multivariable analysis as they were all encompassed in the treatment summary variable. Patients who were ≥50 years old had a worse OS than patients <50 years old (HR: 2.79; 95%CI: 1.89–4.1; *p* < 0.001). Patients who had a tumor size >6 cm had a worse OS than patients with a tumor size <3 cm (HR: 2.07; 95%CI: 1.23–3.5; *p* < 0.01). Patients who received no treatment (HR: 7.20; 95%CI: 2.26–22.9; *p* < 0.001), CT (HR: 7.96; 95%CI: 1.75–36.3; *p* < 0.01), CT + RT (HR: 4.02; 95%CI: 1.40–11.5; *p* < 0.01), or RT (HR: 7.27; 95%CI: 1.91–27.7; *p* < 0.01) had significantly worse OS compared to patients who received GTR alone. Although sex and tumor location were significant during the univariate analysis, they lost their significance during the multivariable analysis. We made a forest plot representing our multivariable analysis (Figure 2). KMC survival plots were made for OS, surgical treatment, RT, and CT (Figure 3A–D) as well as sex, age, tumor size, and tumor location (Figure 4A–D).

We then constructed a nomogram using sex, age, tumor location, tumor size, and treatment summary to calculate 5- and 10-year survival probability (Figure 5). We also made a web-based nomogram for patients diagnosed with, and clinicians treating ENB to determine individual patient-specific survival.

We next tested our nomograms’ validity by plotting observed versus predicted 5-year survival which had a concordance index (c-index) of 0.733 ± 0.041. After, we constructed an ROC curve to obtain an AUC of 0.749 (Figure 6A,B).

Internal validation was then performed using bootstrap resampling (1000 replicates) to account for model optimism. The performance of each bootstrap model was evaluated on both the bootstrap sample (to obtain bootstrap performance) and the original study cohort (to obtain test performance). Optimism was calculated as the difference between the bootstrap and test performance for each re-sample. The average optimism across all 1000 re-samples was subtracted from the apparent AUC of the original model, yielding a bias-corrected estimate of discriminative ability. Following internal validation, AUC became 0.714.

We then performed temporal validation as a proxy for external validation. We did so by chronologically dividing the data by year of diagnosis into an earlier (diagnosed ≤ 2017, *n* = 346, AUC = 0.755) and a later (diagnosed ≥ 2018, *n* = 111, AUC = 0.786) cohort. This split represents an 80% training and 20% testing cohort and serves to evaluate if model performance is stable across different diagnostic eras.

## 4. Discussion

Our study found a median and mean OS of 13.6 years and 12.83 years, respectively, as well as 1-, 2-, 3-, 5- and 10-year survival probabilities of 89.6%, 84.7%. 80.3%. 74.7% and 58.2%, respectively, (Table 1). These survival numbers are in the middle compared to what is commonly cited in the literature.

Meta-analyses by Dulguerov et al., and Skolnick et al., with 390 and 49 patients found that 5-year OS was 45% and 52%, respectively, while a cohort of 49 patients by Morita et al. found a relatively higher 5-year OS of 69% [6,14,15]. Tajudeen et al. found a 5- and 10- year OS of 61% and 50%, respectively, in a large cohort of 281 patients [16]. A meta-analysis of 97 patients by Elkon et al. noted a crude survival of 65.4% from 6 months to 32 years depending on follow up with OS at 3 and 5 years being Kadish-stage dependent [17].

Small retrospective cohorts by Zeng et al. and Rosenthal et al. found a 77% and 93% 5-year OS in 64 and 31, respectively [18,19]. Additional small retrospective cohorts by Eden et al., Bacher et al. and Dias et al. confirmed this finding with 40, 39, and 35 patients who averaged 5-year OS rates of 78%, 88%, and 55% as well as 10-year OS rates of 71%, 69%, and 46%, respectively [7,8,20]. A small retrospective cohort of 17 patients by Kadish and colleagues had a 62% OS at 1 year [12]. Marinelli and colleagues found that in a large retrospective study of 118 patients with ENB who had distant metastases, OS was 54% at 6 months [9]. This highlights the large variability in OS with 5-year OS ranging from 45% to 93% [6,7,8,14,15,16,18,19,20].

### 4.1. Sex

Our study found that ENB was more common in males (57%), and that males had a significantly worse OS than females.

Large meta-analyses by Vuong et al. (733 patients) and Tajudeen et al. (281 patients) found that males were more likely to be diagnosed with ENB (57% and 55%) and also had a significantly worse OS [16,21].

However, Dias et al. and Morita et al. found that although males were more likely to be diagnosed with ENB (55% and 57%), sex did not impact OS [8,15]. Malouf and colleagues retrospectively investigated 44 ENB patients with a 1:1 sex ratio, and found that there was no difference between 5-year OS in males (62%) versus females (63%) [3]. Marinelli and colleagues found that sex did not impact OS [9].

Dulguerov & Calcaterra conducted a small retrospective cohort study of 24 patients distributed equally based on sex and found that females had a non-significantly worse progression-free survival compared to males, but did not investigate OS [13]. This suggests that the true effect of sex on OS may not be fully understood.

### 4.2. Age

Our study had a mean age of diagnosis of 53.91 ± 16.82 years old with a median of 55 years old. This is concurrent with most of the literature that notes a peak age of onset of 50–60 years old. We also note that being ≥50 years old significantly worsens OS.

Tajudeen and colleagues had a mean age of diagnosis of 52 years old and found that advanced age significantly worsened OS [16]. Dulguerov & Calcaterra found a mean and median age of diagnosis to be 40 and 41 years old, respectively, with a significantly worse prognosis (undefined) in patients >50 compared to <50 years old [13]. Marinelli and colleagues confirmed that older age significantly impacts OS [9].

Morita and colleagues found a mean age of diagnosis of 48 years old with bimodal peaks at ages 20–30 and 50–60 but found that age (3–37, 39–54, 55–62 years old) did not impact OS [15]. Bachar and colleagues supported this finding with a mean age of diagnosis of 50 years old and age being non-impactful to survival [7]. Dias and colleagues found no significant difference between OS of patients under versus over 50 years old [8]. Malouf and colleagues’ study showed a lower median age of diagnosis at 43 years old, and they found a non-significant difference in 5-year OS in patients over (59%) versus under (66%) 46 years old [3]. Based on the literature and our findings, age impacts OS but the extent to which it does so is not fully known.

### 4.3. Race + Tumor Size + Tumor Location

Our study noted that race did not impact OS. While race was not widely explored in many ENB studies, a study of Vuong et al. (733 patients), Tajudeen et al. (281 patients) and Sharma et al. (561 patients) also noted that race did not impact OS [16,21,22].

Our analysis revealed that tumor size, both 3.0–6.0 cm and >6.0 cm impacted OS compared to <3.0 cm. Other studies focused largely on tumor staging and grading rather than size. Our study demonstrated that tumors located in the paranasal sinuses resulted in significantly worse OS compared to those in the nasopharynx and nasal cavity. Although ENB is typically confined to the nasal and paranasal cavities, four non-CNS cases were present. This unusual presentation likely reflects extensive tumor burden, which correlates with a poor prognosis and decreased OS. Due to a low sample size, we excluded these patients from our analysis to prevent obscuring interpretation of our nomogram.

While we did not look specifically at tumors based on the Kadish grading system, stage A of the system is tumors confined to the nasal cavity while stage B includes tumors extending into the paranasal sinus [12]. Ours is slightly different as we looked at primary tumor site not extension/invasion of the tumor. Most studies in the literature note some inadequacies in the Kadish grading system, with some combining stages A and B [13,20,23] and others adding stage D for distant metastases, dubbed the modified Kadish [9,15,16]. Of those who use Kadish staging, a higher stage is correlated with worse outcomes including OS, disease free survival and local recurrence [3,7,8,9,13,15,17,19]. Although not explored here, this highlights the importance of considering grading and staging when determining survival.

### 4.4. Treatment

Our study found that GTR was a significantly better treatment option than CT, CT + RT, RT, STR + CT, GTR + CT, STR + CT + RT and no treatment at all. We found that OS was similar between patients who received GTR and STR.

Malouf et al., Vuong et al., Tajudeen et al., and Zeng et al. noted that treatment with either surgery or RT alone significantly improved OS compared to no treatment [3,16,19,21]. Malouf et al. and Vuong et al. noted that CT alone compared to no treatment significantly improved OS [3,21]. Zeng et al. further specified that surgery (type unspecified) and RT combined lead to significantly better OS (84%) compared to surgery (51%) and RT (38%) alone [19]. However, Dulguerov & Calcaterra noted that 2 of 5 patients who were treated with RT alone died within 3.5 years, while 10 of 11 patients who received RT and surgery combined were alive and disease free for longer (years unspecified) [13].

Skolnik and colleagues found that 5-year OS was 64% for surgery, 38% for RT, and 50% for combined therapy, although significance calculations were not noted [14]. Morita et al. found that treatment with GTR and post-operative RT has a significantly better OS than STR alone or STR and RT combined [15].

Bachar et al., Elkon et al. and Eden et al. note the importance of Kadish staging with regards to treatment [7,17,20]. Elkon et al. suggests that RT and RT + surgery are equally effective for early stages (A-B) with later stages (C) being mostly palliative [17].

Marinelli et al. found that patients who received CT with either surgery and/or RT had the highest OS (95%) compared to patients who received monotherapy (56%) or no treatment (33%) at 6 months [9].

We also noted that STR and GTR had comparable OS. The literature either supports this finding or denotes the treatment modality as surgery and does not explore the difference between GTR and STR [13,14,17,19]. One potential reason for this, is that different surgical approaches can be applied to tumor removal. Open craniofacial resection and transfacial surgery can both be used for tumors spreading to the orbit or palate [24]. However, the literature notes that endoscopic endonasal approaches are often superior if negative margins can be obtained, as they avoid facial incision which can lead to facial scars or bone defects [4,24,25]. Overall, tumor size, location, proximity to other anatomic structures and patient comorbidities must be considered when electing the correct surgical approach. Considering there is no agreed upon standard-of-care, treatment needs to be tailored to the individual patient but one must note the importance of surgery (STR or GTR) as a treatment modality [24].

### 4.5. Nomogram

Nomograms offer a predicted survival based on demographic, pathological and clinical factors, serving as vital clinical tools to assist with cancer research and treatment. Using nomograms, physicians can provide individualized treatment plans based on patient-specific factors. Based on an individual variables’ significance in predicting the outcome of interest (overall survival), nomograms use a point-based system to assign weight to each factor [26,27].

Normally, only significant variables are included in nomograms to ensure high clinical utility. However, non-significant demographic variables are frequently included as they can help to improve patient stratification. Nomograms can handle these variables by assigning lower points to the non-significant versus significant variables [28].

Our nomogram was built with a large patient cohort (*n* = 457), but additional data can improve the model’s current predictability. To validate our nomogram, we used a predicted versus observed 5-year survival plot, and an ROC curve. Predictive accuracy was measured by a c-index to gain an understanding of our model’s ability to correctly discriminate between outcomes (alive versus dead). Our value of 0.733 ± 0.041 highlights an acceptable predictive value which is supported by an AUC of 0.749, indicating an acceptable distinguishing ability of survivors versus non-survivors of ENB [29,30].

Based on patients’ sex, age, tumor size, and tumor location, clinicians can determine the best treatment for patients and simultaneously counsel patients on their predicted OS based on a given treatment. Our web-based nomogram is the first to be done for ENB and provides a quick and easy method for providers to help guide clinic-decision making.

### 4.6. Limitations and Future Directions

The SEER database has limitations inherent to its large, retrospective design. These include a lack of (1) treatment timing, which makes it challenging to determine the chronological order of interventions; (2) histopathological data, such as Hyams grade, mitotic activity, necrosis, rosette formation, all of which are important for ENB diagnosis and could provide further patient stratification to improve model predictability; (3) tumor recurrence rate, repeated surgery number and disease progression, all of which could be impactful for morbidity, mortality and quality of life. The lack of these variables impacts the predictive accuracy of the nomogram.

Additionally, excluding 483 patients due to an unknown tumor size reduced our sample size. However, despite missing data points, this national registry allows us to analyze a highly representative cohort, maximizing both the power and generalizability of our study; something single-center studies cannot accomplish. Overall, these factors along with the acceptable discrimination ability of our nomogram should be considered during the clinical decision-making process.

## 5. Conclusions

In this study, we examined the most significant prognostic factors for determining OS in 941 patients with ENB. We found that sex, age, tumor size, tumor location, and treatment modality all play important roles in OS. We also provided KMC curves for our significant univariate variables as well as a forest plot and nomogram for our significant multivariable analysis. Moreover, our web-based nomogram offers a personalized calculation of patient-specific survival based on age, sex, tumor size and location and treatment modality. This is the first web-based nomogram developed for ENB. Future studies could improve upon the predictive accuracy of our model by performing external validation with prospective multi-center data and by including additional variables like histopathologic grading, treatment timing, and long-term recurrence data.

## Figures and Tables

**Figure 1:**
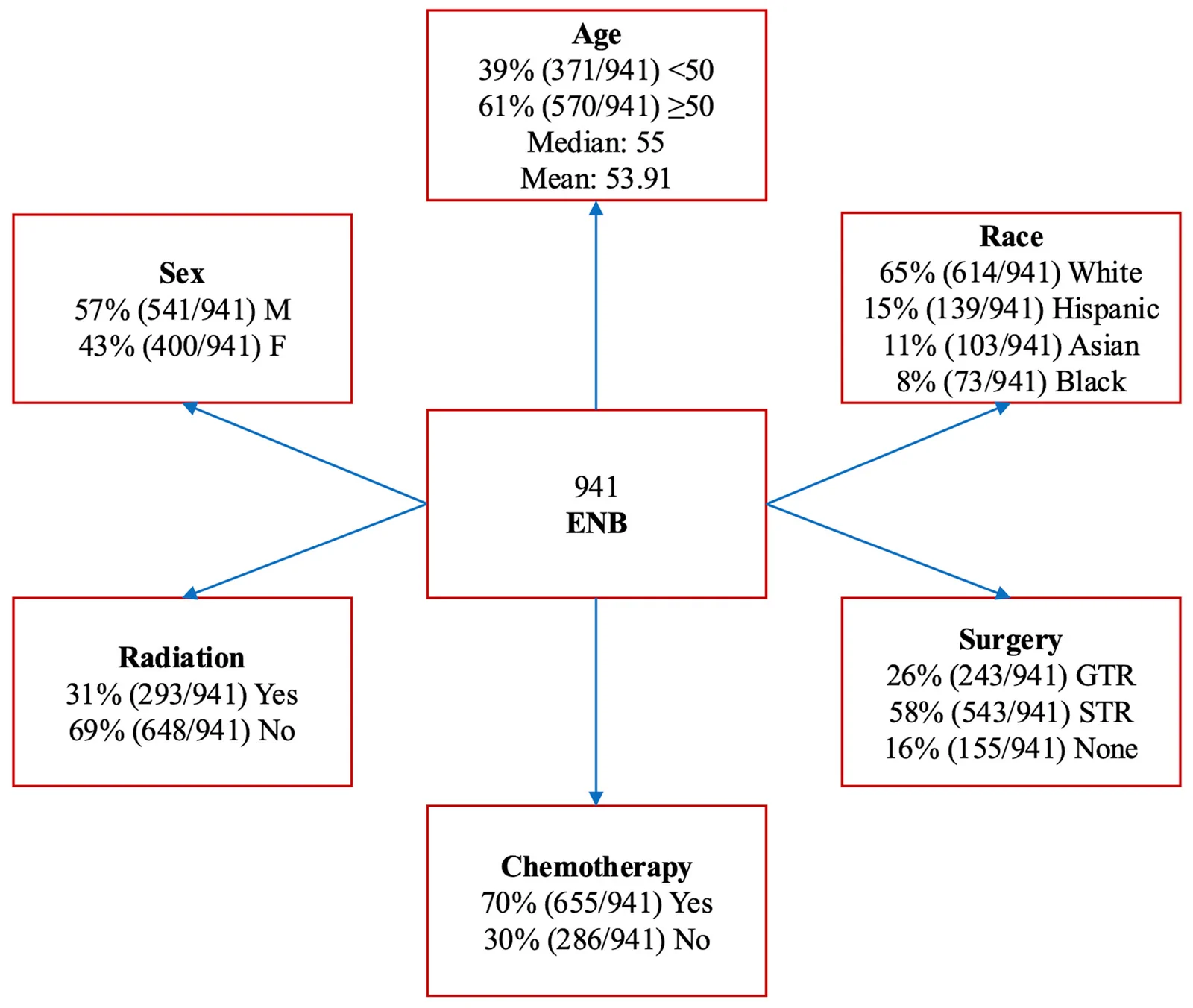
Nine hundred and forty-one ENB patients. Of these patients most were male, white, over the age of 50, and received CT > STR > RT = GTR.

**Figure 2:**
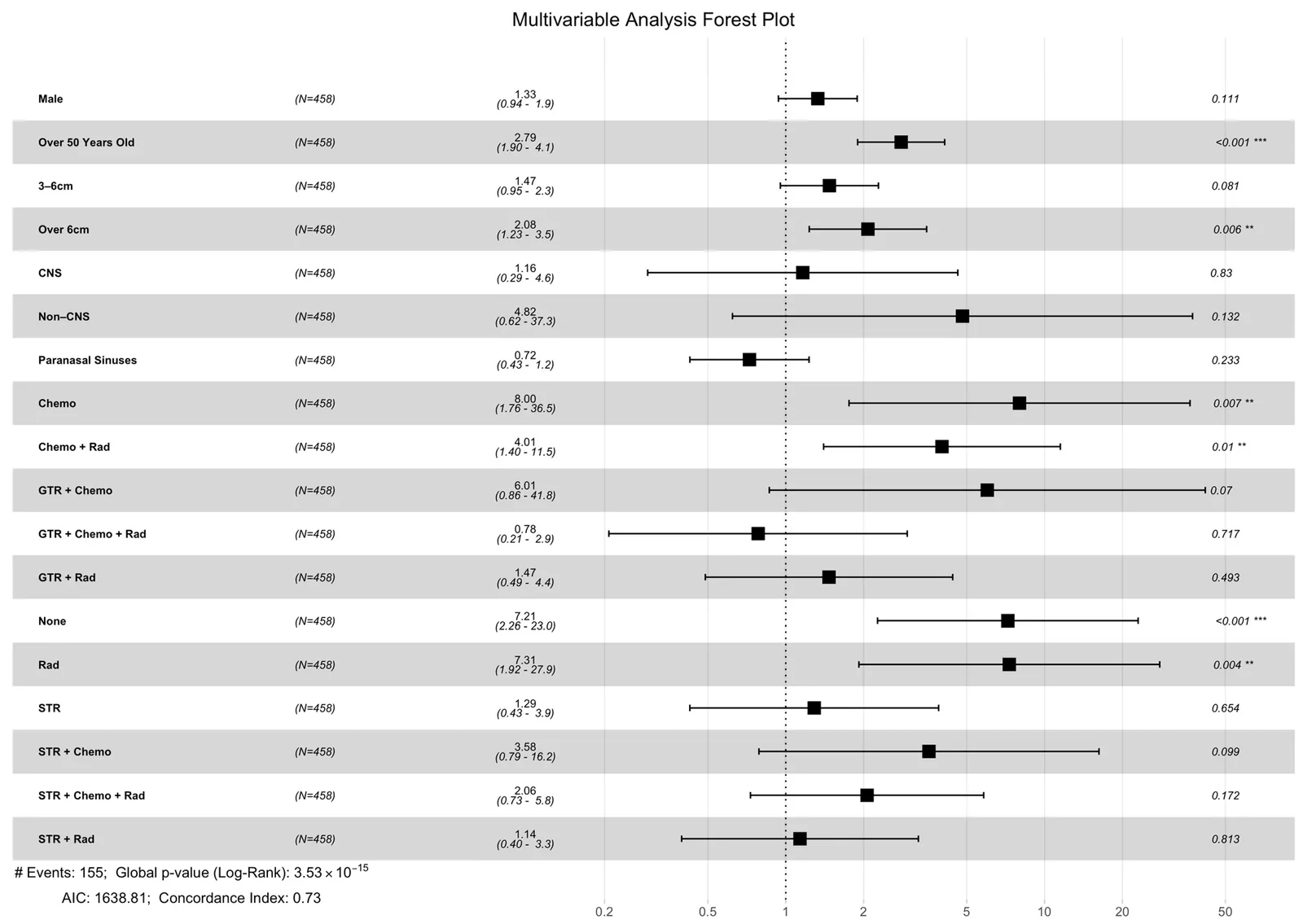
Forest plot of multivariable Cox proportional hazards ratio for sex, age, tumor size, tumor location, and treatment. CNS—central nervous system, Chemo—chemotherapy, Rad—radiation therapy, GTR—gross total resection, STR—subtotal resection. *** *p* < 0.001; ** *p* < 0.01.

**Figure 3:**
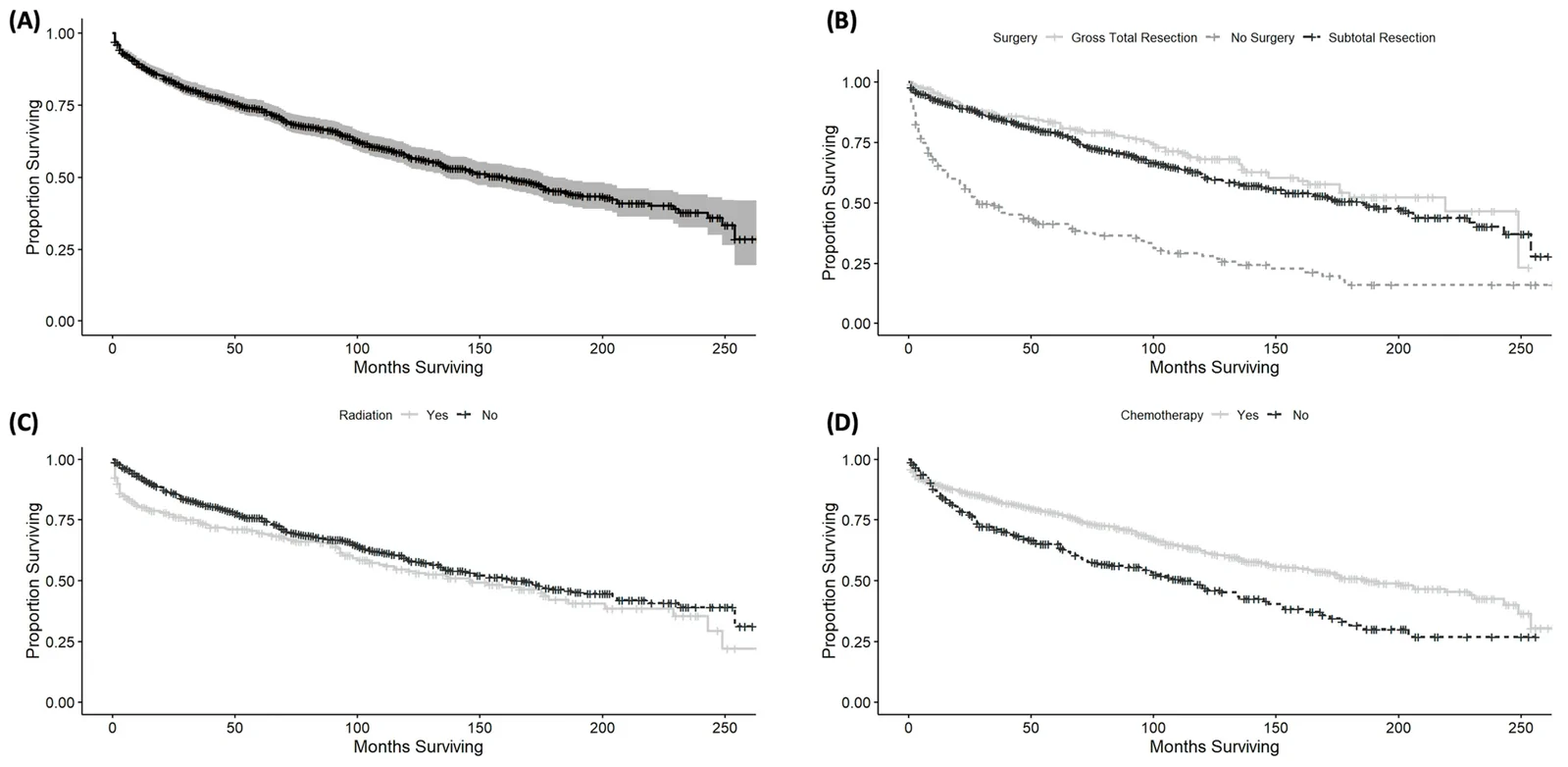
(**A**). Kaplan–Meier survival curve for total population of patients with ENB. (**B**) Kaplan–Meier survival curves categorized by surgical treatment. (**C**) Kaplan–Meier survival curves categorized by radiation treatment. (**D**) Kaplan–Meier survival curves categorized by chemotherapy treatment.

**Figure 4:**
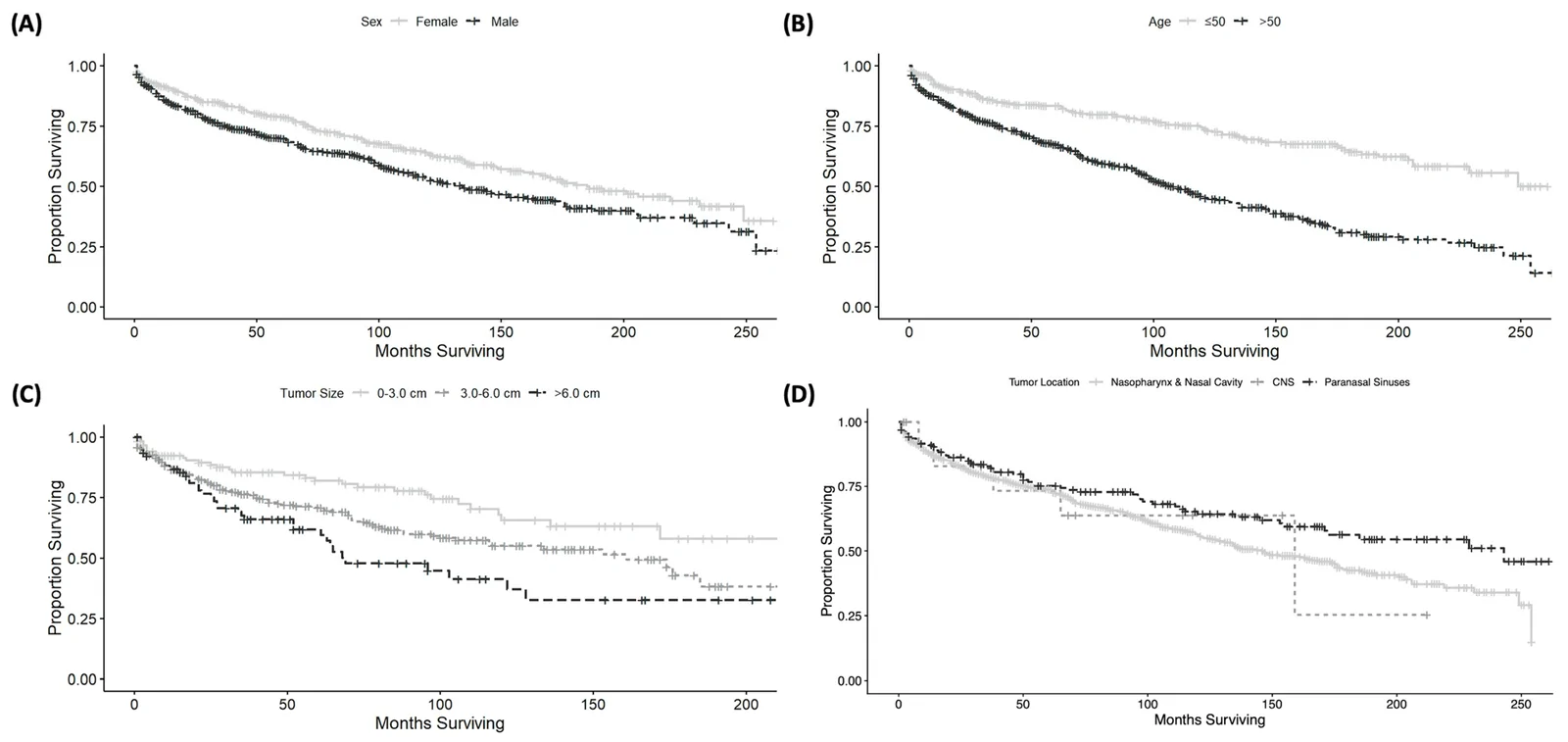
(**A**) Kaplan–Meier survival curves categorized by patient sex. (**B**) Kaplan–Meier survival curves categorized by patient age. (**C**) Kaplan–Meier survival curves categorized by tumor size. (**D**) Kaplan–Meier survival curves categorized by tumor location.

**Figure 5:**
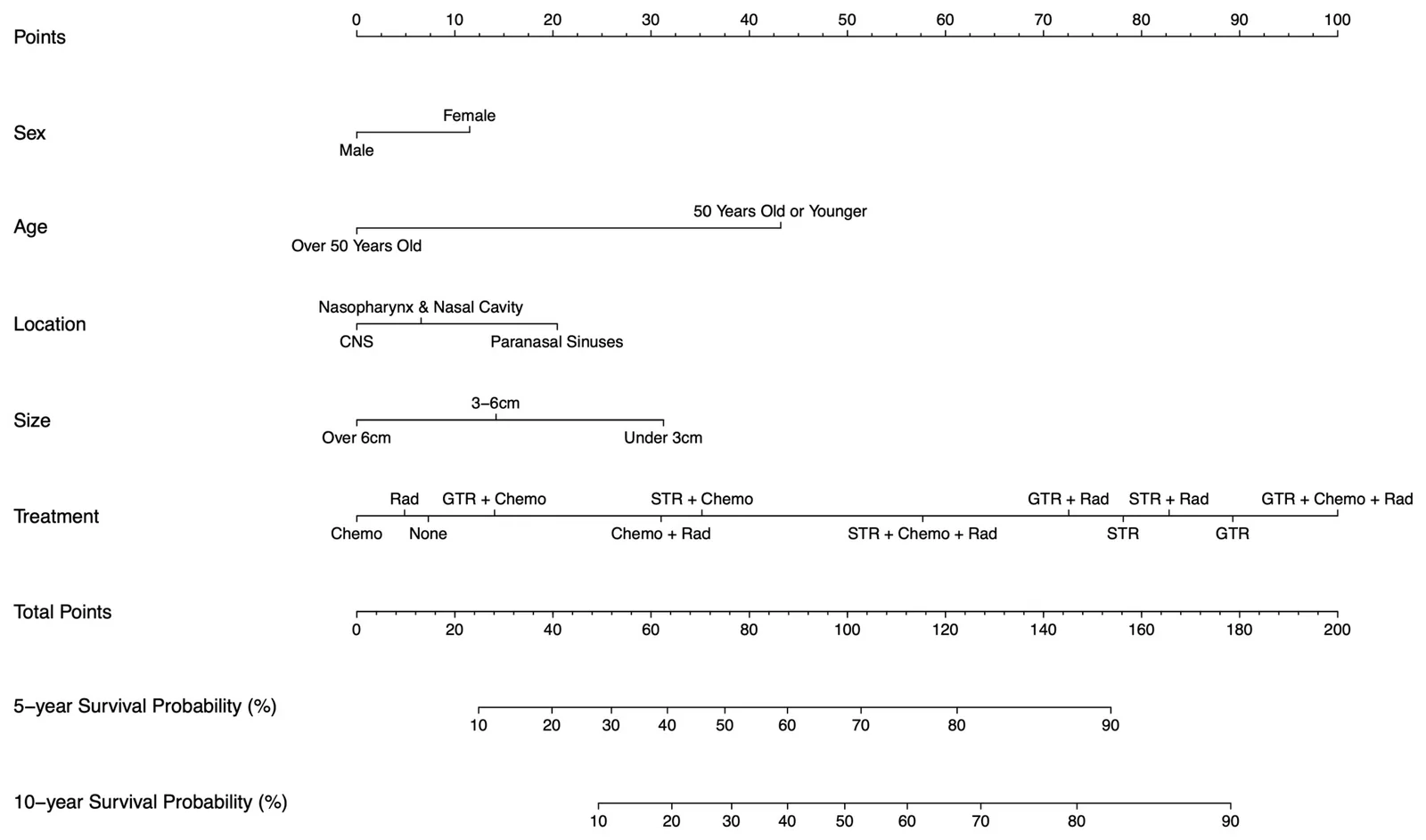
Nomogram demonstrating median survival time as well as 5- and 10-year survival probability based on sex, age, tumor location, tumor size, and treatment summary. CNS—central nervous system, Chemo—chemotherapy, Rad—radiation therapy, GTR—gross total resection, STR—subtotal resection.

**Figure 6:**
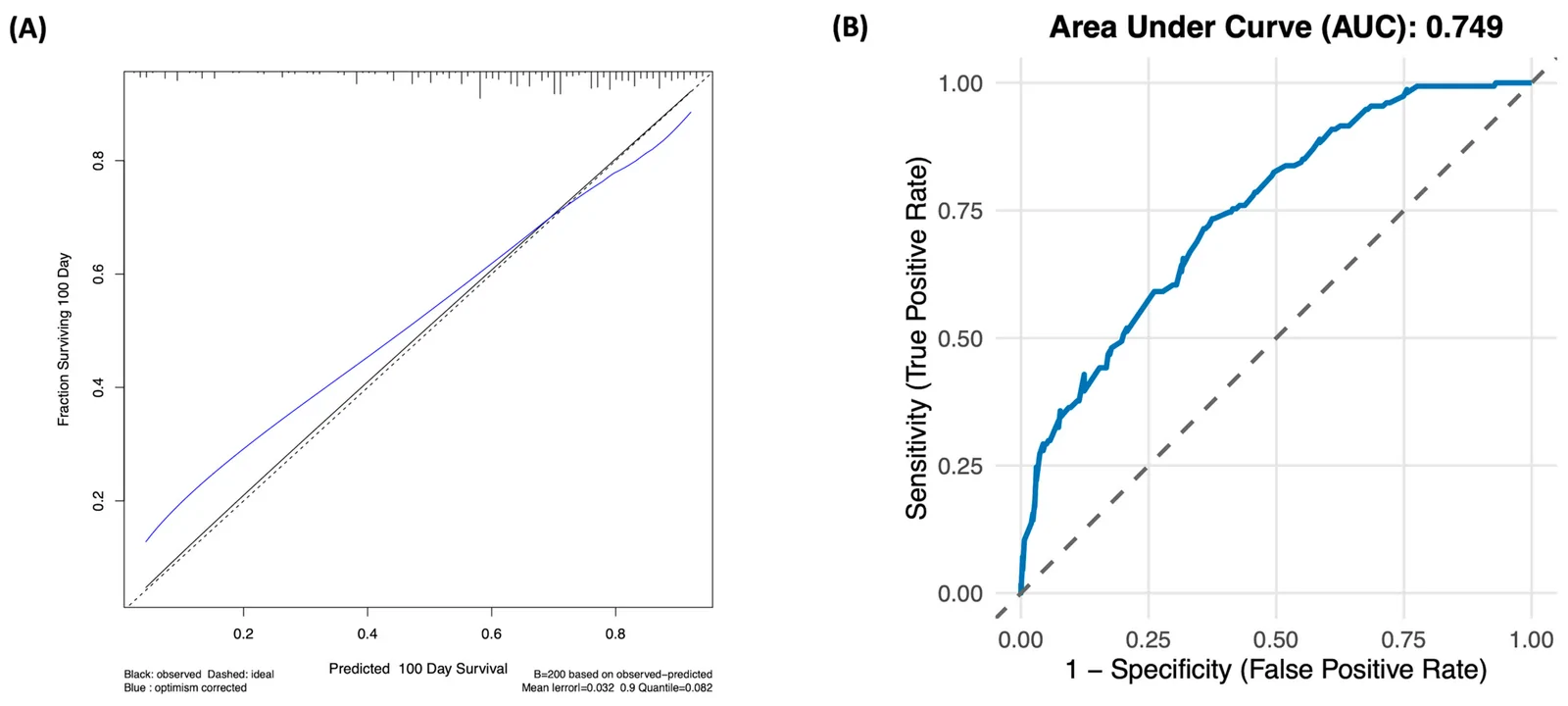
ENB predictive nomogram (**A**) calibration plot with the black solid line representing observed survival, the blue solid line representing optimism-corrected survival, and the black dashed line representing ideal survival and (**B**) a receiver operator curve (ROC) plot with area under the curve of 0.749 and C-index of 0.733 ± 0.041.

**Table 1:** Overall Survival.

Time	Survival
1 year	89.6%
2 years	84.7%
3 years	80.3%
5 years	74.7%
10 years	58.2%
Median	13.6 years (95% CI 11.5–15.5 years)
Mean	12.83 years

**Table 2:** Univariate analysis of overall survival.

Category	Subcategory	Patients (*n* = 941)	Events (*n* = 365)	HR	[95% CI]	*p*
Sex	Female	400	141	reference		0.004
	Male	541	224	1.36	[1.10–1.68]	***
Age	0–50	371	97	reference		5.00 × 10^−16^
	50–100	570	268	2.5	[1.98–3.16]	***
Race	White	614	242	reference		0.5
	Hispanic	139	47	0.88	[0.65–1.21]	
	AI/AN	5	2	0.89	[0.22–3.60]	
	API	103	40	0.99	[0.71–1.40]	
	Black	73	34	1.35	[0.94–1.94]	
Year of Diagnosis	2000–2010	437	237	reference		0.9
	2010–2020	504	128	0.98	[0.78–1.24]	
Tumor Size (cm)	<3	120	29	reference		0.001
	3.0–6.0	260	90	1.65	[1.08–2.51]	*
	>6.0	78	36	2.47	[1.51–4.03]	***
Tumor Location	Nasopharynx and nasal cavity	767	301	reference		0.005
	Central nervous system	14	5	0.95	[0.39–2.31]	
	Paranasal sinus	156	55	0.69	[0.52–0.93]	*
Treatment Summary	GTR	59	14	reference		2.00 × 10^−16^
	CT	10	8	10.7	[4.49–25.7]	***
	CT + RT	63	45	4.33	[2.38–7.90]	***
	GTR + CT	4	3	6.76	[1.94–23.6]	**
	GTR + CT + RT	57	19	1.61	[0.81–3.22]	
	GTR + RT	123	35	1.23	[0.67–2.30]	
	None/unknown/other/refused	65	39	4.49	[2.43–8.28]	***
	RT	17	13	7.51	[3.52–16.0]	***
	STR	142	49	1.59	[0.87–2.87]	
	STR + CT	13	8	4.47	[1.87–10.7]	***
	STR + CT + RT	139	56	2.05	[1.14–3.68]	*
	STR + RT	249	76	1.23	[0.70–2.18]	

*** *p* < 0.001; ** *p* < 0.01; * *p* < 0.05.

## Data Availability

We used de-identified information that is publicly available via the SEER database which is published by NCI.

## Abbreviations

The following abbreviations are used in this manuscript:

ENB	Esthesioneuroblastoma
SEER	Surveillance, Epidemiology and End Result
NCI	National Cancer Institute’s
ICD-O-3	International Classification for Oncology, version 3
OS	Overall Survival
KMC	Kaplan–Meier curves
GTR	Gross total resection
STR	Subtotal resection
CT	Chemotherapy
RT	Radiotherapy
AI/AN	American Indian/Alaska Native
API	Asian or Pacific Islander
CNS	Central nervous system
C-index	Concordance index
ROC	Receiver operator characteristic
AUC	Area under the curve
